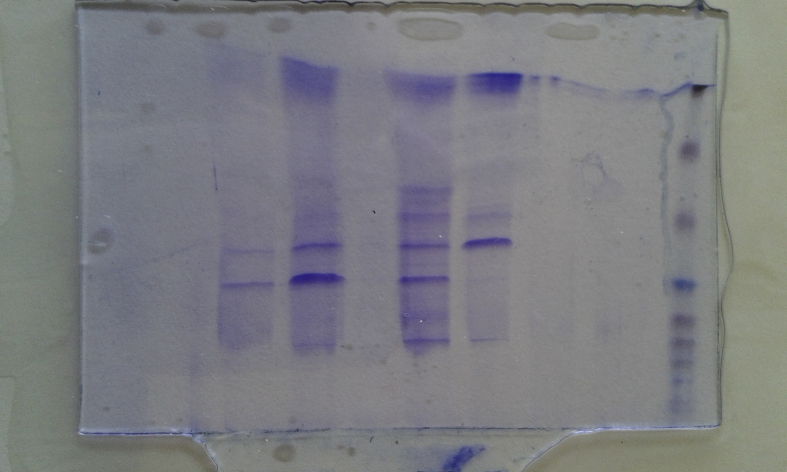# Partial purification and characterization of chitinase produced by *Bacillus licheniformis* B307

**DOI:** 10.1016/j.heliyon.2020.e03858

**Published:** 2020-05-03

**Authors:** Yasser Akeed, Faiza Atrash, Walid Naffaa

**Affiliations:** a Plant Biology Department, Faculty of Science, University of Damascus, Syria; b Plant Protection Department, Faculty of Agriculture, University of Damascus, 30621 Damascus, Syria

**Keywords:** Biochemistry, Biotechnology, Microbiology, Molecular biology, Plant biology, Chitinase, *Bacillus licheniformis*, Optimization, Submerged culture, Purification

## Abstract

The optimal conditions required for chitinase production from *Bacillus licheniformis* B307 strain, obtained from Syrian soil, were studied. Optimization experiments were carried out under submerged fermentation conditions, and colloidal chitin was the source of carbon. Luria broth medium supplied with 0.5% colloidal chitin was the optimum medium for chitinase production. The maximum chitinase yield was obtained at 30 °C, pH6, incubation time 14 days, and 150 rpm. The optimum chitinase activity was achieved at 60 °C and pH6. The chitinase activity with unmodified medium was 1.9 U/mL which then enhanced about eight folds to reach 14.2 U/mL under optimized submerged fermentation conditions. An extracellular chitinase of *Bacillus licheniformis* B307 was partially purified using ammonium sulfate precipitation followed by concentration with various sizes of concentrator tubes. The chitinase was partially purified 8.24 fold and specific enzyme activity increased 2.08 fold (2 U/mg). Sodium dodecyl sulfate polyacrylamide gel electrophoresis (SDS-PAGE) of partial purified chitinase exhibited a molecular weight (*M*_r_) near to 36 and 42kDa. These results make it possible to invest in this strain to produce chitinase to be used as antifungal, food additives and other applications.

## 1 Introduction

Insoluble chitin is linear polymer of β-1,4-Nacetylglucosamine (GlcNAc), and it considers from the most naturally abundant polysaccharide and it is one of the major component of most fungal cell walls (Sadfi et al., 2001). Therefore, degradation of chitin is important step for recycling it as a nutrients in the nature. Hydrolytic enzymes Chitinases (EC 3.2.1.14) break down glycosidic bonds in chitin. Hence, Chitinases that are produced by microorganisms have crucial roles and wide range of applications. Chitinases can be used for bio-conversion of chitin into useful products in biotechnology, waste management, pharmaceuticals, biomedical applications, single-cell protein, isolation of protoplasts from fungi and yeast, drug delivery, and enzyme industry (Kumar et al., 2018; Patel et al., 2017; Patel and Goyal, 2017). In addition, there are many other important potential applications of chitinase, for instance it can be recruited as potential biocontrol agents against many fungal pathogens (Downing and Thomson, 2000; Gomaa, 2012), and possible future applications as food additives to increase shelf life (Hamid et al., 2013).

*Bacillus* species are one of the largest sources of bioactive natural compounds (Emmert and Handelsman, 1999), they form endospores and produce large amount of secondary metabolites, and classified as safe andbeneficial to grops and environment (Shoda, 2000). Several species of *Bacillus* have shown chitinolytic activities, such as *B. pumilus* (Agarwal et al., 2017), *B. subtilis* (Ashwini and Srividya, 2014; Narasimhan and Shivakumar, 2012; Saber et al., 2015), *B. licheniformis* strain LHH100 (Laribi-Habchi et al., 2015), *B. licheniformis* (Xiao et al., 2009)*, B. thuringiensis* (Gomaa, 2012).

The production of extracellular chitinase, which has gained more attention across the world (Wang et al., 2006), can be enhanced by improvement of culture medium composition and fermentation conditions. Improved production conditions are critical to achieve optimal yield, productivity, and to reduce production costs (Abdel-Fattah et al., 2005). Currently, submerged fermentation (SmF) methods, which involve in production of enzymes by microorganisms in a liquid nutrient medium, are widely practiced to decrease infection and increase the possibility of greater yield of enzymes. Compared to solid state fermentation, SmF facilitates handling and controlling environmental factors such as pH and temperature. The fermentation medium optimization can be carried out through several strategies. One of them is the classical technique “one-factor-at-a-time” for chitinase production (Singh et al., 2017; Sukalkar et al., 2018).

This study aimed to optimize the culture and fermentation conditions of chitinase produced by *Bacillus licheniformis* B307 strain, that has chitinolytic and antifungal activity, by using SmF method. Partial purification for extracellular chitinase from the crude enzyme extract was also performed.

## 2 Materials and methods

### 2.1 Microorganism

*Bacillus licheniformis* B307 was isolated from Syrian soil (Salamiyah, N:34^⁰^ 57^⁰^ 48.5^⁰^) and it showed chitinolytic and antifungal activity against *Botrytis cinerea* (Akeed et al., 2019). The strain was maintained on nutrient agar (NA) at +4 °C and 20% glycerol at -80 °C. The inoculum of *Bacillus* strain was prepared by inoculating 20 mL of sterilized nutrient broth (NB) in 100 mL flask with loop full of pure *Bacillus licheniformis* B307 and incubated overnight at 30 °C and 200 rpm until the optical density at 600nm reached 0.15 which equals to 2 × 10^8^ CFU/mL (Ammoneh et al., 2014).

### 2.2 Colloidal chitin preparation

The colloidal chitin was prepared according to the method described by Rodriguez-Kabana et al. (1983) with addition of 20g of chitin in 500mL of concentrated HCl. The chitin was added to the acid with vigorous stirring at 25 °C until it dissolved (1.5–2 h). The mixture was incubated in a water bath at 37 °C with gentle stirring till the mixture became transparent (0.5 h). The mixture then was filtered by using glass wool to remove impurities and undissolved particles. The filtrate was added to 5L cooled distilled water with stirring for 0.5 h, then it was placed at 4 °C without stirring for 24 h. The precipitate was collected and washed with distilled water by centrifuging until pH reached 5–6, and stored in the dark at 4 °C until used. 10 mL of colloidal chitin was taken and dried at 80 °C for 24 h to calculate the dry weight and determine the concentration of chitin.

### 2.3 Chitinase assay

Chitinolytic activity was estimated by dinitrosalicylic acid (DNS) method using colloidal chitin as a substrate according to the method described by Sadfi et al. (2001) with some modification. The reaction matrix had 0.5 mL of 1% colloidal chitin suspension in 0.1 M sodium acetate buffer pH5, 0.4 mL of enzyme solution. The mixture then incubated 30 min at 50 °C and then the reaction was terminated by 1mL DNS (NaOH 10 g/L, dinitrosalicylic acid C_7_H_4_N_2_O_7_ 10 g/L, phenol C_6_H_6_O 2 g/L, and adding Na_2_SO_3_0.05g/100mL and sodium potassium tartrate C_4_H_4_KNaO_6_._4_H_2_O 20 g/100mL when using). The color of the mixture was developed by incubating it for 10 min at 100 °C. Centrifugation was performed at 7500 ×*g* for 10 min, then the supernatant adsorption was measured at 540nm. A standard curve was plotted using N-acetyl glucosamine (NAG, Sigma). One unit (U) of chitinase activity represent the amount of released enzyme of 1μmol N-acetyl glucosamine of colloidal chitin per min under reaction conditions.

### 2.4 Optimization of chitinase production

SmF experiments were performed in 100 mL flasks that contain 20 mL of the culture medium, and inoculated with 1 mL of bacterial inoculum. Various chemical and physical parameters were studied and their effect on production of the enzyme was recorded.

### 2.5 Effect of medium composition on chitinase production

Different types of media were implemented for optimal production of chitinase including NB medium, consists of (g/L): 1 beef extract, 2 yeast extract, 5 peptone, 5 sodium chloride; LB medium, consists of (g/L): 10tryptone, 5 yeast extract, 5 sodium chloride; *M medium*, consists of (g/L): 0.5 yeast extract, 1 (NH_4_)_2_SO_4_, 1 KH_2_PO_4_, 0.3 MgSO_4_._7_H_2_O; and Y medium, consists of (g/L): 5casein, 5 galactose, 1 KH_2_PO_4_, 0.3 MgSO_4_._7_H_2_O; Z medium, consists of (g/L):0.5 yeast extract, 2 Na_2_HPO_4_, 1 K_2_HPO_4_, 1 NH_4_Cl, 0.5 CaCl_2_._2_H_2_O, 0.5 NaCl, 0.5 MgSO_4_._7_H2O. Colloidal chitin was added to each of the mentioned media to a final concentration of 0.5%. All the culture media were adjusted at pH 7. The cultivation experiments were carried out in shaking incubator 150 rpm, 30 °C, for 5 days.

### 2.6 Effect of colloidal chitin concentration on chitinase production

Production medium was prepared with different concentrations of colloidal chitin (0.1, 0.3, 0.5, 0.7, 0.9 and 1%), and incubated under shaking conditions (150 rpm) at 30 °C.

### 2.7 Effect of incubation temperature on chitinase production

To determine the optimum incubation temperature for chitinase production, cultivation was carried out at different incubation temperatures ranged from 20 to 40 °C with variation of 5 °C.

### 2.8 Effect of pH on chitinase production

The influence of pH on chitinase production was monitored, where the pH of production mediums were adjusted before sterilization, from pH4 to pH9 at intervals of 1 unit, using NaOH 1M and HCl 1M.

### 2.9 Effect of incubation time on chitinase production

The optimum incubation time was determined for optimal chitinase production, inoculated flasks were incubated in a rotary shaker at 150 rpm and 30 °C. Every 24 h the culture filtrate was harvested and the enzyme activity was measured for 15 days of incubation time.

### 2.10 Effect of agitation speed on chitinase production

Optimum agitation speed was determined by incubating the production medium in Erlenmeyer flasks 100 mL at different agitation speed in shaker incubator ranging from 50 to 200 rpm with interval unit of 50 rpm.

## 3 Enzyme characterization

### 3.1 Effects of both pH and temperature on chitinase activity

To determine the optimal temperature for chitinase activity, the reaction mixtures incubated at different temperatures ranged from 20 to 80 °C and then assayed the enzyme's activity under standard conditions using colloidal chitin as a substrate. Also, optimal pH for chitinase activity was determined by measuring the activity at 60 °C within a range of pH from 4 to 9 using sodium-acetate buffer (pH4.0–6.0), sodium-phosphate buffer (pH7.0–8.0) and glycine-NaOH buffer (pH 9.0).

### 3.2 Partial purification of chitinase

The crude enzyme was partially purified from the culture supernatant using saturated ammonium sulfate (SAS). The precipitation was carried out at 4 °C in stirring conditions using different percentage of SASs 45%, 55%, 65%, 75%, and 85%. Precipitates were collected by centrifugation at 11500 ×*g* for 10 min at 4 °C and dissolved in a volume of buffer sodium-acetate pH6 equal to the size of crude enzyme. Protein determination in each fraction was performed according to Bradford (1976) and bovine serum albumin was used as a standard. An estimate of the chitinase activity was carried out according to the assay method described above. The optimum specific chitinase activity at a specific concentration of ammonium sulfate reflected the best concentration to attain maximum enzyme recovery. The fraction, which gave the highest specific chitinase activity, was taken to second partial purification using various concentrator tubes (Sartorius, vivaspin 2) MWCO 10kDa (MWCO: Molecular Weight Cut-off), MWCO 30kDa, and MWCO 50kDa. The best precipitation product was taken, first separated using 10kDa membrane and centrifugation 2500 ×*g* for 30 min, then the supernatant was separated using 30kDa membrane and centrifugation 2500 ×*g* for 30 min, after that the supernatant was separated using 50KDa membrane and 2500 ×*g* for 30 min finally the total protein and enzyme activity were determined for every fractions, and each were also subjected to electrophoresis on 12% polyacrylamide gel. The gel was stained using 0.5% Coomassie Brilliant Blue R250 (Sigma).

### 3.3 Statistical analysis

STATISTIC program (version 6 Stat soft, Inc. 2003) was used for statistical analyses at 5% level (P = 0.05). The data was subjected to analysis of variance (ANOVA; Tukey's HSD test) for the determination of differences in means between treatments. The percentages were analyzed by applying normal approximation test (analysis of proportions). All the experiments were repeated three times.

## 4 Results and discussion

### 4.1 Obtaining a new strain producing chitinases and antifungal activity

Chitinase has been previously isolated and characterized from a repertoire of *Bacillus* species (Khan et al., 2018; Oyeleye and Normi, 2018; Veliz et al., 2017). However, it is important to obtain chitinases with new characterization, and meet the application needs of these enzymes. Indeed, the chitinase that has been obtained in this study from the strain B307 has shown new properties compared to the ones in the literature (Table 1). In addition, the strain B307 possesses an antifungal property attributed to the chitinase activity which may broaden the use of this strain to biocontrol of some plant pathogens (Akeed et al., 2019). The strain B307 showed enzyme activity 14 ± 1.075 U/mL with temperature tolerance up to 60 °C. Comparing to other strains (Table 1) the strain B307 from this study has an increase in enzyme activity at 60 °C which makes it a potential candidate for industry.

**Table 1:** Comparison between chitinase activity from *B. licheniformis* B307 and other works.

Strain	Optimal condition for chitinase activity		Chitinase activity U/mL	Reference
	pH	Temperature (°C)		
*Bacillus licheniformis* A2	5	70	25.3	Khiyami and Masmali (2008)
*Bacillus licheniformis* B307	6	60	14 ± 1.075	Our work
*Bacillus licheniformis* JP2	6	60	0.05	Keliat et al. (2016)
*Bacillus licheniformis* Mb-2	6	70	3.5	Toharisman et al. (2005)
*Bacillus cereus* SV1	7	55	0.08	Ghorbel-Bellaaj et al. (2011)
*B. licheniformis*	8	40	23	Gomaa (2012)

### 4.2 Effect of medium composition on chitinase production

**Figure 1:**
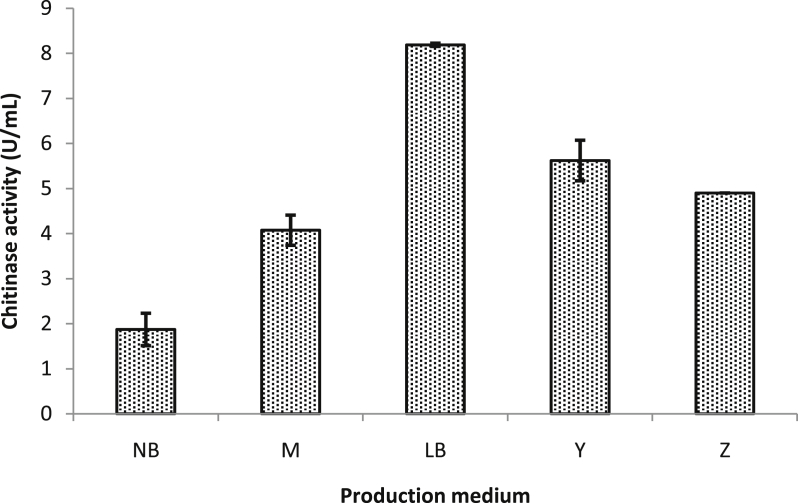
Effect of medium composition on chitinase production (30 °C and 5 days incubation with agitation 150rpm).

The components of the medium have an essential role in the growth and metabolism of microorganisms. Therefore, different broth media have been tested to optimize the culture medium for chitinase production. The results clearly showed that maximum chitinase production of 8.2 ± 0.035 U∖mL was observed when LB medium was used (Figure 1). In contrast, NB medium showed minimum chitinase yield (1.9 ± 0.35 U∖mL) (DF = 4, F-value = 178.256, P˂0.0001). It is known that the LB nutrient medium is commonly used in the cultivation of many species of bacteria because it permits fast and good growth yields for them, and the addition of colloidal chitin to this medium has stimulated the developing bacteria to produce chitinase. Similar works reported that colloidal chitin in LB has yielded maximum chitinase production by *Bacillus subtilis*, from all the tested media, LB with colloidal chitin showed also more productive in both *Aeromonas hydrophila* and *Aeromonas* punctate strains (Karunya, 2011; Kuddus and Ahmad, 2013).

### 4.3 Effect of colloidal chitin concentration on chitinase production

**Figure 2:**
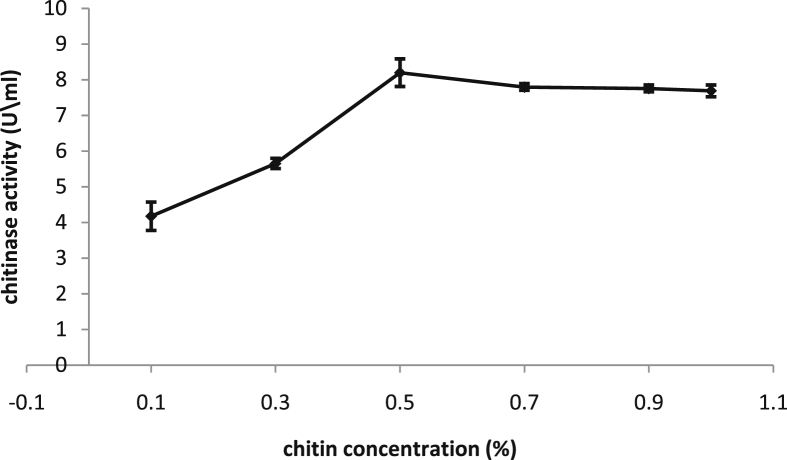
Effect of colloidal chitin concentration on chitinase production by *B. licheniformis* B307.

Production medium optimization is an essential step not only to maximize the yield and productivity, but also to minimize the production costs (Singh et al., 2017). Therefore, several concentrations of colloidal chitin were tested. Results showed that colloidal chitin concentration influenced the production of chitinase by *B. licheniformis* B307. There were obvious coloration between enzyme production and colloidal chitin concentration where the maximum enzyme production of 8,19 ± 0.655U∖mL (DF = 5, F-value = 40.287, P˂0.0001) was observed when 0.5% of colloidal chitin was used (Figure 2). However, no significant increase in enzyme yield beyond 0.5% was noticed. These results are in agreement with earlier findings of chitin which is a vital factor in inducing high chitinase production from microorganisms (Soiuza et al., 2005). Wen et al., (2002) reported that the addition of 0.5% colloidal chitin or more has induced the maximum chitinase production in *Bacillus* sp. NCTV2, as well, Abirami et al., (2016) reported that colloidal chitin concentration between 0.5-1% enhanced the chitinase production considerably by *B. licheniformis* SSCL10. In addition, the highest chitinase production by *B. pumilus* was obtained by using a medium supplemented with 0.5% chitin (Bhattacharya et al., 2016).

### 4.4 Effect of incubation temperature on chitinase production

**Figure 3:**
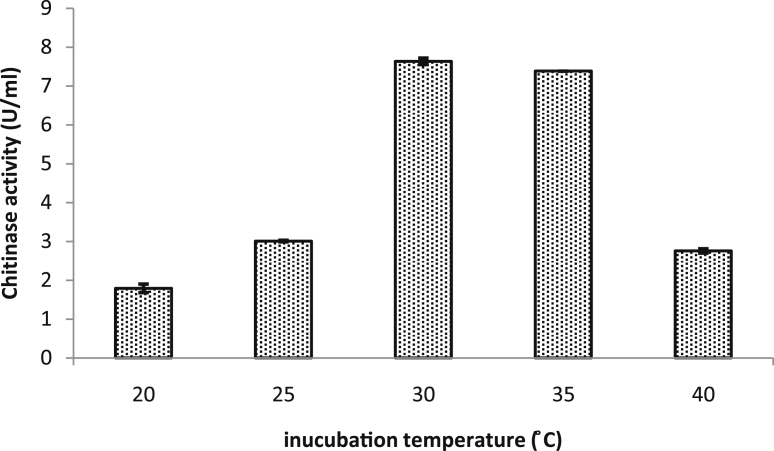
Effect of incubation temperature on chitinase production by *B. licheniformis* B307. Error bars represent the standard error of three replicates.

The changes in incubation temperature within the studied range (20–40 °C) showed significant effect on the chitinase yield produced by *B. licheniformis* strain B307. The maximum production was found to be 7.6 ± 0.145 and 7.3 U/mL (DF = 4, F-value = 1706.641, P˂0.0001) at 30 °C and 35 °C respectively, while significant reduction in the production was observed when the temperature increased or decreased from the previous values (Figure 3). Temperature has an impact on various biological processes, therefore the growth of bacteria and the production of enzymes are influenced by the incubation temperature modification. Narasimhan and Shivakumar (2012) reported maximum chitinase production from *B. subtilis* at 30 °C, while a number of researchers reported that the optimum temperature for production of chitinase from *Bacillus licheniformis*, *B. laterosporus*, *B. pumilus* and *B. subtilis* is 35 °C (Jholapara et al., 2013; Shanmugaiah et al., 2008; Bhattacharya et al., 2016; Karunya, 2011).

### 4.5 Effect of pH on chitinase production

**Figure 4:**
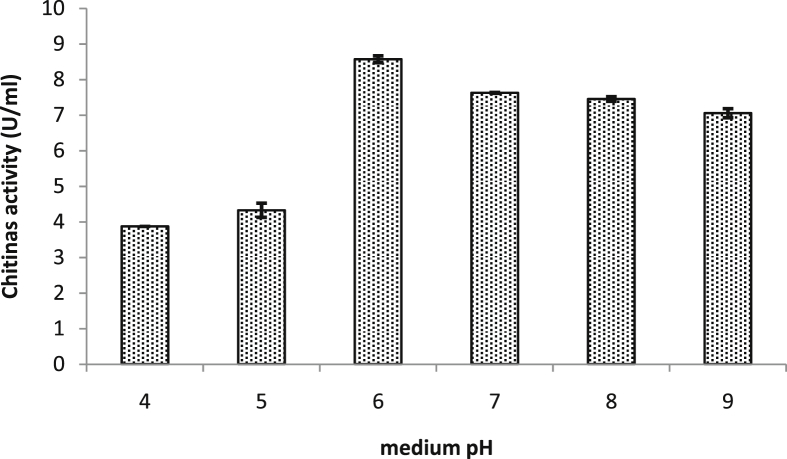
Effect of initial pH of the fermentation media on chitinase production by *B. licheniformis* B307. Error bars represent the standard error of three replicates.

The effect of different initial pH of fermentation medium on the yield of chitinase produced by *B. licheniformis* B307 was tested. The maximum chitinase production was found to be at pH6 with the activity of 8.56 ± 0.165U∖mL, while the minimum chitinase activity 3.87 U∖mL (DF = 5, F-value = 319.445, P˂0.0001) was observed at pH4. However, yield at pH7 to pH9 has not decreased significantly (Figure 4). Culture medium pH has a great effect on microbe growth and metabolism (Pan et al., 2019), therefore the pH should be adjusted when preparing the fermentation medium. Similar researches referred to the variation in the optimum pH for the production of chitinase from *Bacillus*. For example, pH6 was optimal for chitinase production from *Bacillus subtilis* W-118 (Wang et al., 2006), pH7 from *B. thuringiensis* (Gomaa, 2012) and pH8 from *B. laterosprous* (Shanmugaiah et al., 2008).

### 4.6 Effect of incubation time on chitinase production

The maximum chitinase yield was found after incubation for 14 days (9.54 ± 0.04 U/mL) (DF = 14, F-value = 102.24, P˂0.0001), after that, the production began to decline. In many SmF processes, when the production reaches the highest yield, further increase in incubation time can reduce the yield due to the degradation of metabolites (Hao et al., 2012).

### 4.7 Effect of agitation speed on chitinase production

**Figure 5:**
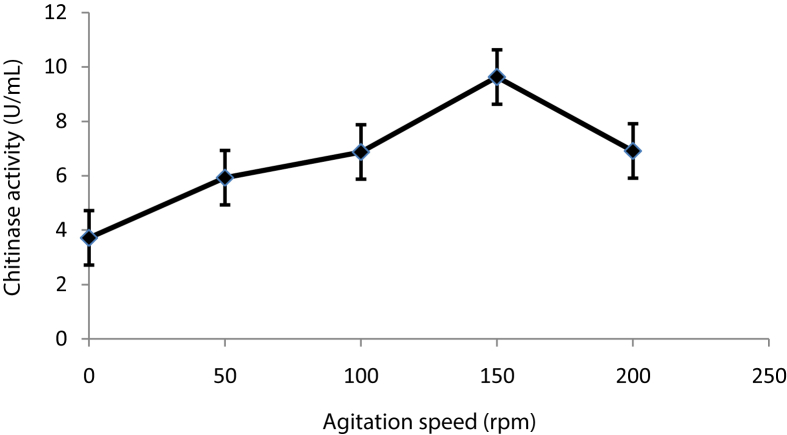
Effect of agitation speed on chitinase production by *B. licheniformis* B307. Error bars represent the standard error of three replicates.

Chitinase production by shaking microorganisms under SmF is better than using static condition (Shivalee et al., 2018). The maximum chitinase production by *B. licheniformis* B307 was observed at agitation speed of 150 rpm (9.63 ± 0.88 U∖mL) (DF = 4, F-value = 41.715, P˂0.0001). The chitinase production was increased as there were increases in the agitation speeds up to 150 rpm, then decreased with increased speed (Figure 5). Similarly, maximum chitinase production by *Bacillus* sp. was found at 150 rpm (Gupta et al., 2017).

### 4.8 Effect of temperature on chitinase activity

**Figure 6:**
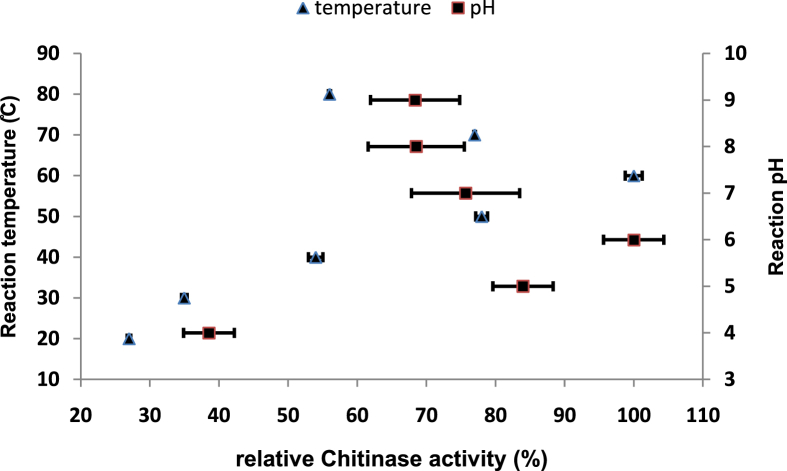
Effect of temperature and different pH values on chitinase activity produced by *B. licheniformis* B307. Error bars represent the standard error of three replicates.

Reaction temperature is one of the determining factors in the effectiveness of enzyme activities. In this study, results showed that the optimum temperature for chitinase activity produced by *B. licheniformis* B307 strain was at 60 °C (10.1 ± 0.215 U/mL) (DF = 6, F-value = 1287, P˂0.0001). So, when the temperature increased or decreased from 60 °C, the activity of chitinase gradually reduced. The relative activity of chitinase was 80% at 50 and 70 °C (Figure 6). In Table 1, the optimal temperature for chitinase activity from *B. licheniformis* B307 was compared to chitinases produced from other strains in similar works.

### 4.9 Effect of pH on chitinase activity

Enzymes activities are affected by changes in the pH value of the reaction, and the point that achieves the highest enzymatic efficiency is known as the optimum pH. Therefore, the pH- relative activity of *B. licheniformis* B307 chitinase was determined in the range pH4.0- pH9.0 (Figure 6), and the optimum pH was found to be at pH6 (14 ± 1.075 U/mL) (DF = 5, F-value = 12.34, P˂0.0002). In Table 1, the optimal pH for chitinase activity from *B. licheniformis* B307 was compared to chitinases produced from other strains in similar works.

### 4.10 Partial purification of chitinase

**Figure 7:**
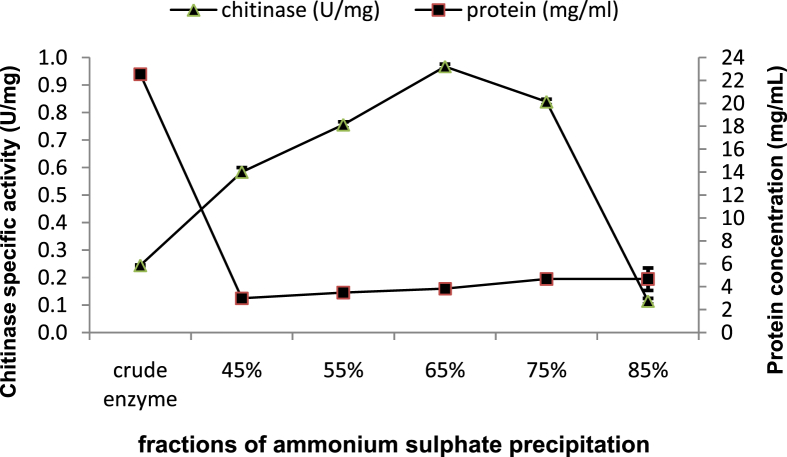
Chitinase activity and protein concentration of various fractions after precipitation crude enzyme with different concentrations of SAS. Error bars represent the standard error of three replicates.

**Figure 8:**
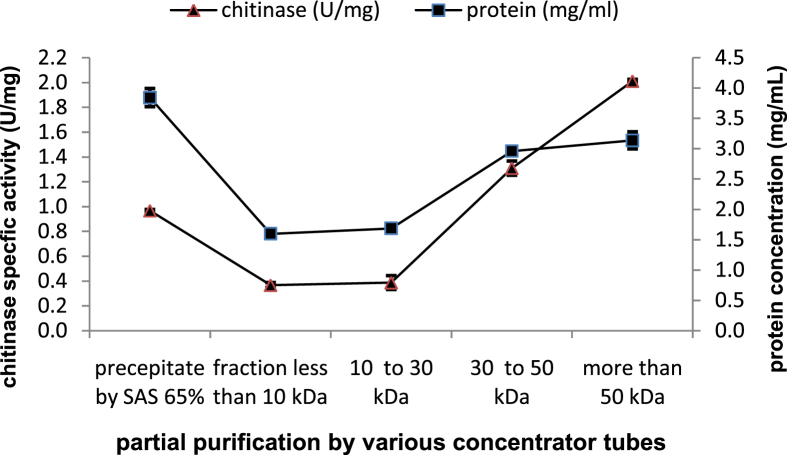
Chitinase activity and protein concentration of various fractions after concentration of 65% SAS by various concentrator tubes. Error bars represent the standard error of three replicates.

**Figure 9:**
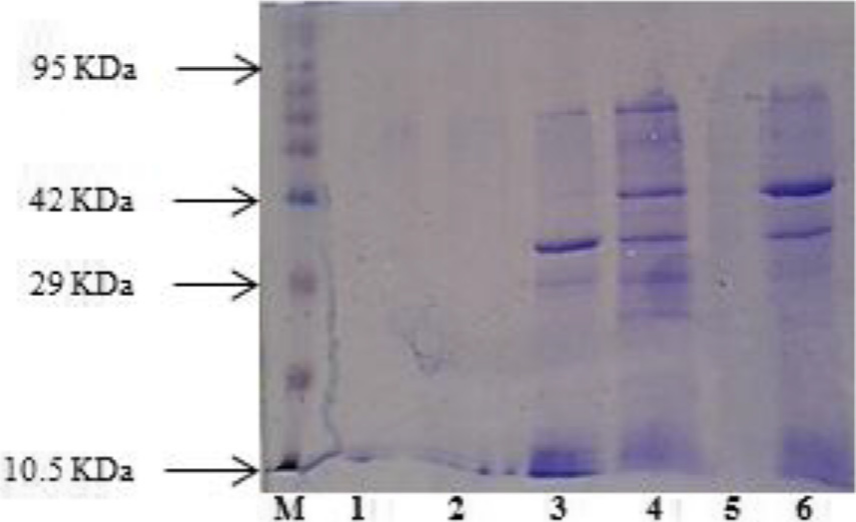
SDS-PAGE analysis for partial purification of chitinase from *Bacillus licheniformis* B307. 1: Flow-throw of MWCO 10kDa tube, 2: fraction between 10kDa and 30kDa, 3: fraction between 30kDa and 50kDa, 4: supernatant of MWCO 50kDa tube, 5: nothing, 6: product of precipitation by 65% SAS. See supplementary Figure 1 for full image.

In the first step, the enzyme was partially purified by precipitation with different concentrations of SAS. Maximum specific enzyme activity 0.97U/mg was observed in the fraction containing 65% of SAS, while in the crude enzyme it was 0.24U/mg (Figure 7). It was found to increase 3.96 fold enzyme activity with 67.52% yield after precipitation with 65% of SAS. After that, in the second step for partial purification of chitinase from *B. licheniformis* B307, the fraction of 65% SAS which gave the highest specific chitinase activity was concentrated by various concentrator tubes. The fraction containing the supernatant of tube MWCO 50kDa showed the highest chitinase specific activity 2U/mg and the specific enzyme activity was increased 2.08 fold with 169% yield from 65% SAS (Figure 8). SDS-PAGE of denatured partial purified chitinase exhibited an *M*_*r*_ near to 36 and 42kDa (Figure 9). Many researches have recorded isolation and purification of chitinases produced by *Bacillus* of different molecular weights: 89, 76, 72, 66, 62, 59, 53, 49, 42, and 36kDa (Kudan and Pichyangkura, 2009; Liu et al., 2010; Takayanagi et al., 1991; Trachuk et al., 1996).

## 5 Conclusion

In this study, the optimal conditions for chitinase production from *B. licheniformis* B307strain were determined using SmF method. A partial characterization was also performed, where the optimum temperature and pH were determined to obtain the best chitinase activity. A partial purification of the extracellular chitinase in the crude extract was also performed, where chitinase was purified to reach 8.24 fold comparing to the crude extract with specific activity 2U/mg for the partial purified enzyme.

Depending on its properties, the chitinase obtained by *B. licheniformis* B307 strain, has a potential use in the industries that have obstacles in using chitinase enzymes at pH6 and/or temperature up to 50–70 °C degrees such as pharmaceuticals, biocontrol and biotechnology applications among others.

## Declarations

### Author contribution statement

Yasser Akeed: Conceived and designed the experiments; Performed the experiments; Analyzed and interpreted the data; Contributed reagents, materials, analysis tools or data; Wrote the paper.

Faiza Atrash: Conceived and designed the experiments; Analyzed and interpreted the data.

Walid Naffaa: Conceived and designed the experiments; Wrote the paper.

### Funding statement

This research did not receive any specific grant from funding agencies in the public, commercial, or not-for-profit sectors.

### Competing interest statement

The authors declare no conflict of interest.

### Additional information

No additional information is available for this paper.

## Figures and Tables

**Supplement _Fig1:**